# Dietary Iron Supplementation Protects Against Growth Restriction and Metabolic Dysfunction‐Associated Steatotic Liver Disease in Perinatal Cadmium‐Exposed Mice

**DOI:** 10.1096/fba.2025-00045

**Published:** 2025-06-17

**Authors:** Rebecca Lichtler, Hannah Klossner, Nikia Smith, Cathrine Hoyo, Michael Cowley

**Affiliations:** ^1^ Department of Biological Sciences North Carolina State University Raleigh North Carolina USA; ^2^ Center for Human Health and the Environment North Carolina State University Raleigh North Carolina USA; ^3^ Genetics Program North Carolina State University Raleigh North Carolina USA; ^4^ Toxicology Program North Carolina State University Raleigh North Carolina USA

**Keywords:** anemia, cadmium, dietary supplements, iron deficiency, liver diseases, maternal exposure, pregnancy

## Abstract

Iron (Fe)‐deficiency (ID) and Fe‐deficiency anemia (IDA) are highly prevalent conditions and are of particular concern to maternal–child health. ID and IDA are typically linked to nutritional deficiencies, but maternal exposure to heavy metals including cadmium (Cd) also leads to offspring with low levels of circulating Fe. Another comorbidity of ID and IDA is metabolic dysfunction‐associated steatotic liver disease (MASLD), a liver condition characterized by lipid accumulation and fibrosis. We have previously shown that maternal Cd exposure also leads to the development of MASLD in offspring. We hypothesized that providing Fe fortification would prevent Cd‐induced ID, which would in turn rescue offspring from growth restriction and MASLD. To test this, virgin dams were exposed to 30 ppm of cadmium chloride (CdCl_2_) in their drinking water during the preconception, gestation, and lactation periods. Fe fortification was supplied in the form of dietary ferric citrate, which amounted to two (2×) or five times (5×) the normal dietary Fe in standard chow. Our study provides evidence that perinatal Cd exposure does not prevent absorption of supplemental Fe, and that the chosen Fe supplementation dosages are sufficient to prevent Cd‐induced growth restriction, ID, IDA, and MASLD in offspring at postnatal day 21 (PND21). Our findings suggest that Fe supplementation may be a viable therapy to prevent these developmental effects of maternal Cd exposure.

## Graphical abstract

Developmental cadmium (Cd) exposure causes iron deficiency (ID) and impacts growth and metabolism. To test whether iron (Fe) supplementation prevents Cd‐induced effects, female mice were given water and normal chow, or water with Cd in combination with normal chow or chow fortified with 2× or 5× Fe. Offspring of mice exposed to Cd alone developed ID, ID anemia (IDA), restricted growth, and signatures of metabolic dysfunction‐associated steatotic liver disease MASLD. These outcomes were largely rescued by supplemental dietary Fe.

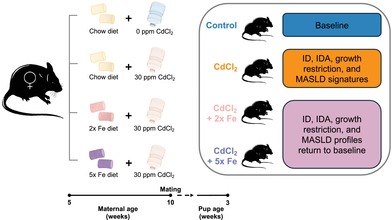

## 1 Introduction

In spite of regulations and guidelines designed to minimize contact with heavy metals, cadmium (Cd) remains present in soil, well water, and consumer products like cigarettes, rechargeable batteries, ceramics, and drinking glasses [1, 2, 3, 4, 5]. Pregnancy is a particularly vulnerable time for Cd exposure due to a programmed increase in uptake of divalent metals [6, 7]. Unlike some developmental toxicants such as alcohol, once Cd enters the body it is not easily excreted; thus, even preconception exposure generates a persistent burden that can impact fetal development [8].

One of the most reproducible effects of cadmium toxicity is iron (Fe) deficiency (ID) and ID anemia (IDA), which can be found in mothers, newborns, or both [9, 10, 11, 12, 13]. Maternal‐child IDA is associated with poor birth outcomes, including prematurity, fetal growth restriction (FGR) and low birth weight (LBW), and even mortality. As the child grows, IDA may persist, leading to stunted growth, attention‐deficit/hyperactivity disorder, cognitive delays, and motor dysfunction [14, 15, 16, 17]. At the population level, signatures of ID and IDA tend to correlate with Cd levels [18, 19, 20, 21]. Because these studies are cross‐sectional in design, there is debate in the scientific community about whether the predominant nature of this relationship is that Cd induces ID or that ID increases the rate of Cd uptake [22]. Several studies have presented evidence in adult mouse models that Cd exposure damages the healthy machinery that absorbs nutritive Fe from the intestines [9, 10]. Until now, it has been unclear if oral Fe supplementation, in this case in the form of dietary fortification, would be able to supersede this phenomenon to provide sufficient physiologically available Fe to return circulating Fe levels to normal.

Cd exposure has also been linked to diseases of the liver [23, 24, 25, 26, 27, 28, 29, 30, 31, 32, 33, 34, 35, 36, 37, 38, 39]. We recently reported our findings that perinatal Cd exposure programs the development of juvenile metabolic dysfunction‐associated steatotic liver disease (MASLD), previously referred to as nonalcoholic fatty liver disease (NAFLD) [31]. MASLD is typically linked to obesity, Western diet, and physical inactivity, among other comorbidities and risk factors [40]. Our findings showed that maternal exposure to Cd through drinking water is sufficient to induce molecular, biochemical, and histological signatures of MASLD at postnatal day 21 (PND21), a timepoint that represents human adolescence [31]. Signs of MASLD are appearing in younger and younger patients, and the latest estimates put the global prevalence of juvenile MASLD at 5%–10%, which presents a significant challenge to the healthcare system [41]. IDA is a commonly observed comorbidity of MASLD, which is typically interpreted as a consequence of MASLD‐related inflammation, although the mechanism has not been experimentally investigated [42, 43, 44].

We designed an experimental mouse model expanding upon our previous studies [12, 31, 45, 46]. Dams were exposed to Cd through their drinking water and, at the same time, received an Fe fortified diet. In this study, we tested the hypothesis that supplementary Fe can rescue perinatal Cd‐induced IDA, growth restriction, and MASLD in juvenile offspring.

## 2 Methods

### 2.1 Exposure Model

**FIGURE 1:**
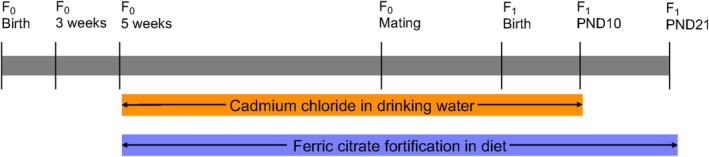
Experimental design. Timeline of *F*_0_ (maternal generation) exposure to cadmium chloride in drinking water and ferric citrate fortification in diet and *F*_1_ (offspring generation) collection.

C57BL/6J mice were housed in the Toxicology Animal Facility and maintained on a 14/10 h light/dark cycle. All mouse work was approved by the North Carolina State University Institutional Animal Care and Use Committee under protocol #22‐162‐B. Drinking water was reverse osmosis (RO) filtered using the Millipore RiOs Essential RO water purification system, and chow was formulated from the AIN‐93G Growing Rodent Diet (Research Diets Inc., D10012G). Starting at 5 weeks of age, maternal Cd burden was generated in virgin females by providing *ad libitum* access to drinking water containing 0 or 30 ppm of CdCl_2_ (Sigma‐Aldrich, 202908) for 5 weeks prior to mating, throughout mating and gestation, and until PND10 (Figure 1). Fe supplementation was delivered through dietary fortification until PND21. Dams were provided with *ad libitum* access to standard chow, containing 212 ppm of ferric citrate, or fortified chow, containing 424 or 1060 ppm of ferric citrate. Four groups were generated from this scheme. Group 1 received normal drinking water and standard AIN‐93G chow (0/1×). Group 2 received drinking water with 30 ppm of CdCl_2_ and standard AIN‐93G chow (30/1×). Groups 3 and 4 received drinking water with 30 ppm of CdCl_2_ and customized AIN‐93G chow with 424 (30/2×) or 1060 (30/5×) ppm of ferric citrate. Fe supplementation dosages were based on previously reported levels used to reverse developmental alcohol toxicity that mimic the intake fold‐change of IDA treatment in human pregnancies [47, 48, 49]. At PND21, offspring were sacrificed by decapitation, and blood and tissues were flash frozen and stored at −80°C until analysis. The right‐median liver lobe was fixed in 4% paraformaldehyde (PFA) overnight, then transferred to 70% ethanol. Plasma was isolated by inhibiting coagulation with EDTA and separating in a refrigerated centrifuge for 15 min at 1000× *g*.

### 2.2 qRT‐PCR

The caudate liver lobe was lysed and homogenized using a Mini Bead Mill homogenizer (VWR, 432‐0366) for 60 s in lysis buffer. RNA was isolated following the NucleoSpin RNA kit manufacturer's protocol (Machery‐Nagel, 740955). Nucleic acid concentration and quality were assessed using the Nanodrop 2000 (Thermo Scientific, ND‐2000). First strand cDNA was synthesized from 1 μg of RNA template by M‐MLV reverse transcriptase (RT) enzyme (Promega, M170A) according to the manufacturer's protocol, using random primers (Promega, C1181). cDNA was diluted by one‐tenth and qPCR was performed using SsoAdvanced Universal SYBR Green Supermix (Bio‐Rad, 1725271). cDNA was amplified and detected using the QuantStudio 3 Real‐time PCR system and Thermo Fisher QuantStudio 5 software. A serial dilution of all pooled samples was used to verify amplification efficiency for each primer set, and PCR product singularity was ensured by inspecting the dissociation curve for each reaction. Relative gene expression was calculated using the previously described 2^−∆∆CT^ method [50]. A full list of primer sequences used can be found in Table S1.

### 2.3 Histology

To visualize neutral lipids, one half of the left liver lobe was flash‐frozen and embedded into optimal cutting temperature (OCT) compound blocks, which were submitted to the University of North Carolina School of Medicine Pathology Services Core (PSC) for processing, sectioning, and staining with Oil Red O (ORO), following established protocols [51]. A portion of the median lobe was fixed in 4% PFA for 24 h and transferred to the PSC in 70% ethanol for paraffin embedding, sectioning, and staining with Sirius Red (SR) to visualize collagen, and hematoxylin and eosin (H&E) to assess gross morphology and Fe deposition, following standard protocols [52].

### 2.4 Total Triacylglyceride (TAG) and Collagen Quantitation

Total hepatic TAGs were quantified with triglycerides reagent (Thermo Fisher, TR22421), using a modified protocol. Briefly, previously frozen liver was digested in an equal volume of 3 M potassium hydroxide in 65% ethanol at 70°C for 1 h with frequent agitation, then for 18 h at room temperature. Digested samples were then neutralized in 2 M Tris–HCl and incubated with Triglycerides Reagent following the manufacturer's recommendations. Total collagen was quantified using the Sensitive Tissue Hydroxyproline assay kit (QuickZyme, QZBtotcol), according to the manufacturer's protocols. Approximately 50 mg of liver tissue input was used for each assay, and both collagen and TAGs were normalized to exact liver mass.

### 2.5 Ferritin and Hemoglobin Assessment

Plasma ferritin was quantified using the Mouse Ferritin ELISA KIT (FTL) (Abcam, ab157713). To measure hemoglobin content, blood samples were briefly warmed to 37°C to reduce viscosity, and hemoglobin content was colorimetrically determined using Drabkin's solution (Sigma‐Aldrich D5941) following a previously established protocol [53].

### 2.6 ICP‐MS

To quantify levels of essential trace metals and Cd, inductively coupled plasma mass spectrometry (ICP‐MS) was performed on blood samples. Previously frozen whole blood was weighed and transferred to Autosampler DigiTUBEs (SCP Science, 010515627). Samples were incubated twice at 95°C for 60 min, first with 8 volumes of 67%–70% PlasmaPure Plus nitric acid (SCP Science, 250038175) and 3 volumes of 32%–35% PlasmaPure Plus hydrochloric acid (SCP Science, 250036115), and second with 8 volumes of PlasmaPure Plus hydrogen peroxide (SCP Science, 250036145). Periodically, several drops of ultrapure water (PURELAB Quest 18.2 MΩ cm water purification system, ELGA LabWater) were added to reconstitute samples. An internal standard containing 10 ppm of germanium, indium, praseodymium, and yttrium was added to each digested sample, and 3% nitric acid was added up to a final volume of 5 mL. A certified custom calibration curve spanning 0–5000 ppb of each reported analyte was generated. ICP‐MS was conducted using a Perkin Elmer Elan DRCII instrument operating in both standard and kinetic energy discrimination (KED) mode to minimize atomic interference. Instrument output was analyzed by adjusting for internal standard levels, and limits of quantification (LOQs) were determined by finding the lowest calibration curve point that did not deviate from the expected analyte concentration by more than 30%. Calculated LOQs can be found in Table S2. Reported isotope concentrations for each sample were normalized to exact blood mass.

### 2.7 Statistical Analysis

All statistical analysis was performed in GraphPad Prism software, version 10 (San Diego, CA, USA). All datasets were first cleaned of statistical outliers using Grubbs' test, then assessed for normality using the D'Agostino & Pearson test if *n* > 7, and the Shapiro–Wilk test for smaller sample sizes. If normality requirements were met, one‐way ANOVA and post hoc comparison with Bonferroni's correction were conducted. If normality requirements were not met, data were analyzed using the Kruskal–Wallis test and Dunn's test for multiple comparisons. Relative risk (RR) *p* values were calculated using Fisher's exact test, and 95% confidence intervals were computed from Koopman asymptotic scores.

## 3 Results

### 3.1 Fe Supplementation Rescues Morphometric Effects of Developmental Cd Exposure

**FIGURE 2:**
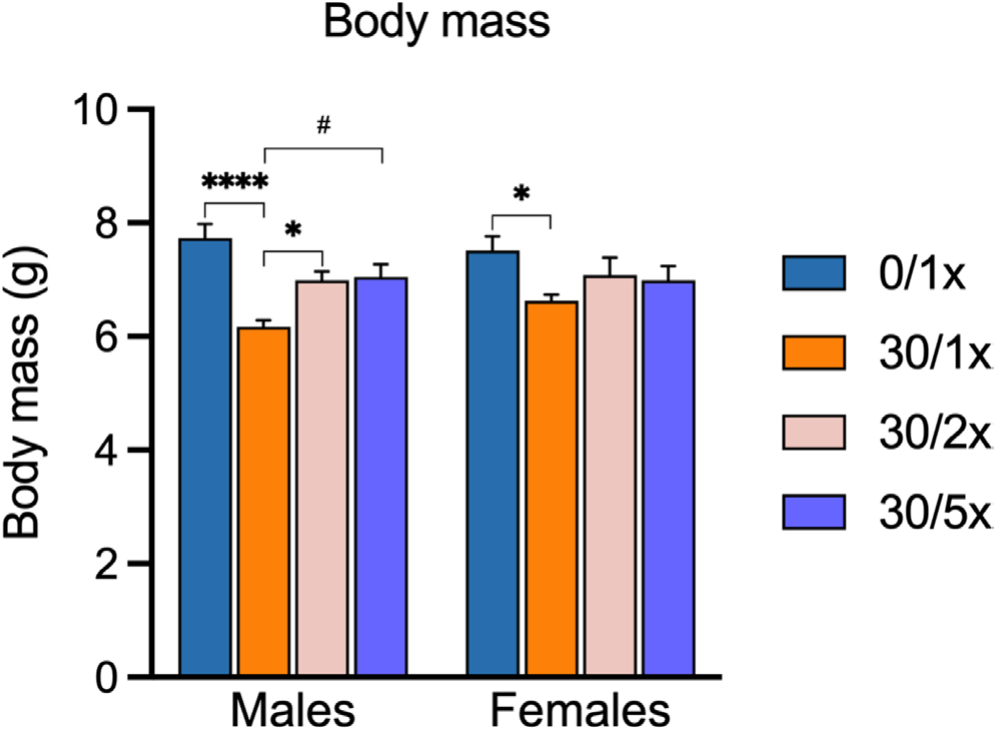
Body mass. Final body mass of pups in grams (g). *p < 0.05; ****p < 0.0001; and ^#^ *p* < 0.1.

Developmental Cd exposure has been shown to cause FGR, LBW, cardiomegaly, liver atrophy, and the brain sparing effect. To determine the efficacy of providing supplemental Fe to rescue these characteristic effects of perinatal Cd exposure, we generated a mouse model wherein the developing mouse was exposed to CdCl_2_ through maternal drinking water and to supplemental Fe via dietary fortification with ferric citrate. A full account of resultant litter and mouse numbers and statistical analysis can be found in File S1. For the purposes of this study, we defined full rescue of any Cd‐induced effects as a scenario where signal in the control group (0/1×) and the indicated Fe supplementation group (30/2× or 30/5×) were both significantly different from the Cd only group (30/1×), but not each other. We defined partial rescue as any other scenario where the Cd only group significantly differed from the control group, and the indicated Fe supplementation group was neither different from the control group nor the Cd only group. Neither food (Figure S1) nor water (Figure S2) intake was affected by oral delivery of Fe or Cd in dams during the 5‐week pre‐gestational treatment period. There was no significant effect of Cd or Fe dose on litter size (Figure S3). Cd alone, represented in the 30/1× group, induced growth restriction in both male and female offspring at PND21 (Figure 2, File S1). Both low‐ (30/2×) and high (30/5×)‐dose Fe supplementation were sufficient to fully return male offspring to normal weight. However, only partial rescue was achieved in female offspring.

**FIGURE 3:**
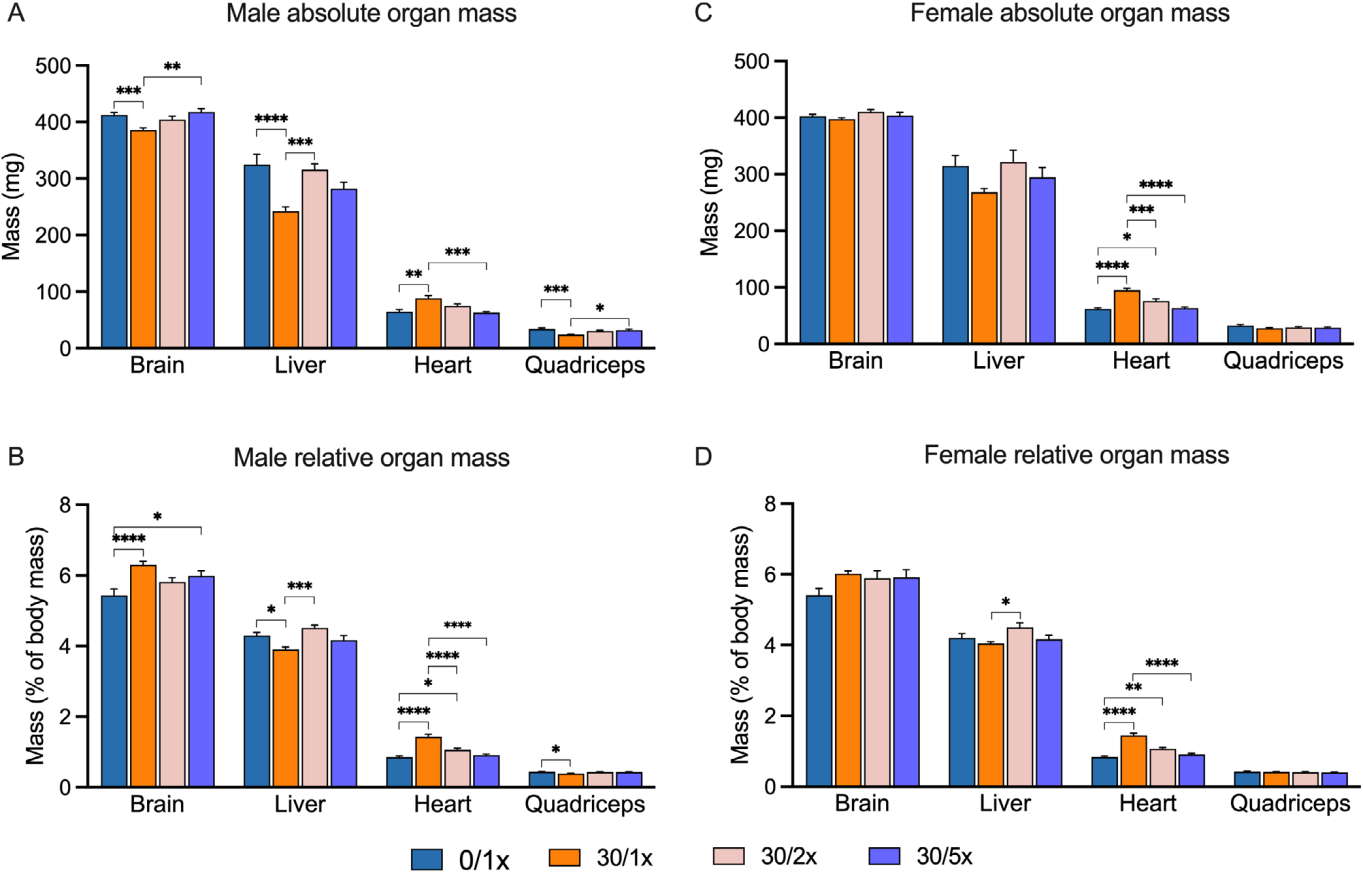
Organ mass. Absolute mass of brain, liver heart, and right quadriceps muscle in milligrams (mg) in (A) males and (C) females. Brain liver, heart, and right quadriceps muscle mass as a percentage of total body mass in (B) males and (D) females. *p < 0.05; **p < 0.01; ***p < 0.001; and ****p < 0.0001.

In males, growth restriction also presented as both absolute and relative quadriceps muscle atrophy, which was at least partially rescued by Fe supplementation (Figure 3A,B, File S1). There was no change in quadriceps mass in female offspring (Figure 3C,D, File S1). Cd caused profound cardiomegaly in both male and female offspring, which was rescued in the 30/2× group and the 30/5× group in a dose‐responsive manner (Figure 3A–D, File S1). Females displayed no other changes in measured organ weights (brain and liver) due to either Cd alone or with either level of Fe supplementation, with the exception of a significant difference between the relative liver mass of the 30/1× and 30/2× groups (Figure 3D, File S1). Male offspring exposed to Cd alone had smaller livers, both absolutely and relatively (Figure 3A,B, File S1). Surprisingly, liver weight was more successfully rescued in the 30/2× group than in the 30/5× group. Males also displayed the brain sparing effect, whereby the brain is partially excluded from Cd‐induced growth restriction because of the importance of the organ's function. The brain weight of mice in the 30/1× group was significantly smaller than that in the 0/1× group, but the brain took up a higher percentage of the total body weight in the same group (Figure 3A,B, File S1). Absolute brain mass returned to normal in the 30/5× group, but relative brain mass did not, and the 30/2× treatment only partially rescued brain phenotypes.

**FIGURE 4:**
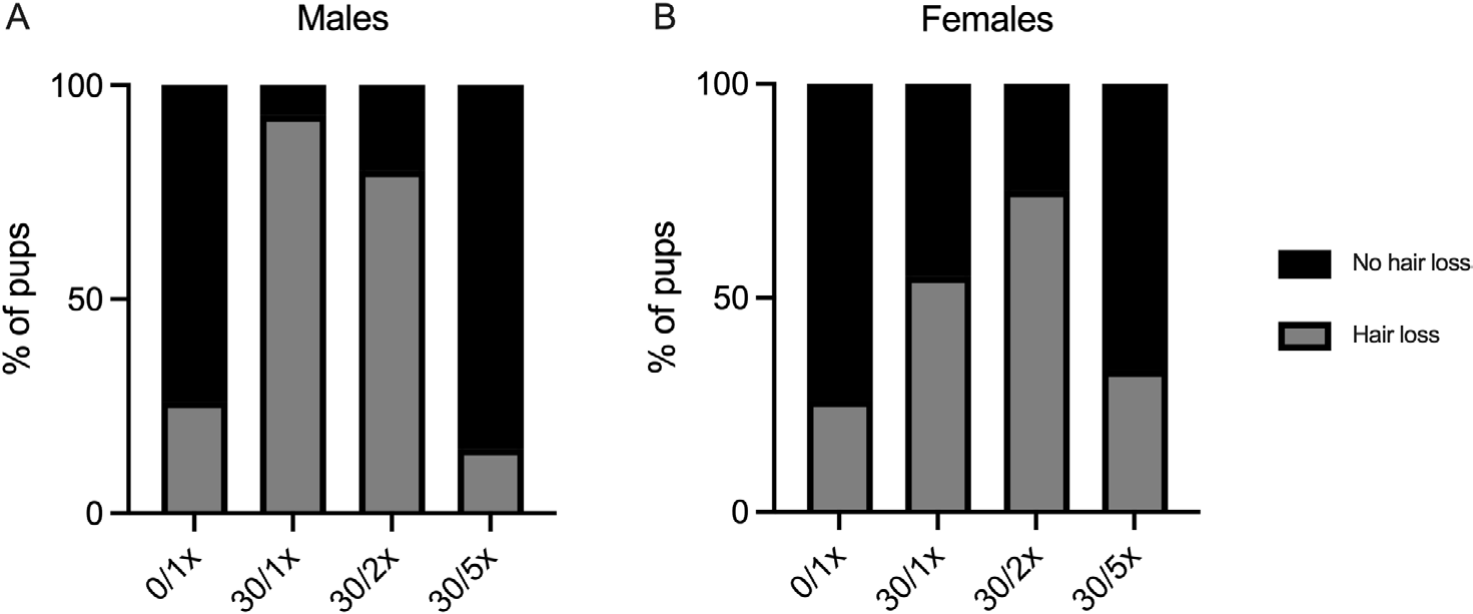
Hair loss. Percentage of (A) male and (B) female pups presenting with hair loss.

Hair loss is a well‐documented feature of many types of poisoning, including heavy metals. 93.3% of male offspring displayed some degree of hair loss due to Cd alone (Figure 4A), representing a RR of 3.5 (1.8–8.0) (Table 1). 2× Fe did not significantly reduce this risk. 80% of 30/2× males exhibited hair loss, representing a RR of 3.0 (1.5–7.0). 5× Fe eliminated the risk of Cd‐induced hair loss. Only 15.4% of males in the 30/5× group lost hair, a statistically similar percentage to the 26.3% in the 0/1× group, determined by the RR of 0.6 (0.1–2.2). In females, Cd alone did not significantly induce hair loss. The RR of the 30/1× group compared to the 0/1× group was 2.1 (0.95–5.0) (Table 1). The 95% CI places the RR just outside the threshold of significance, though there was an upward trend; 55% of the 30/1× group had hair loss, compared to 26.3% of the 0/1× group (Figure 4B). Similar to the males, the 30/2× group displayed an elevated risk—an RR of 2.9 (1.3–6.6)—of hair loss compared to controls, and the 30/5× group displayed full return to the control phenotype, demonstrated by a non‐significant RR of 1.3 (0.5–3.3).

**TABLE 1:** Hair loss.

	Group	Hair loss	No hair loss	RR (95% CI)
Males	0/1×	5	14	Ref.
	30/1×	14	1	**3.5 (1.8–8.0)**
	30/2×	12	3	**3.0 (1.5–7)**
	30/5×	2	11	0.6 (0.1–2.2)
Females	0/1×	5	14	Ref.
	30/1×	11	9	2.1 (0.95–5.0)
	30/2×	9	3	**2.9 (1.3–6.6)**
	30/5×	7	14	1.3 (0.5–3.3)

*Note:* Absolute count of pups presenting with and without hair loss and the relative risk (RR) of hair loss compared to the 0/1× reference group with 95% confidence interval (CI). Significant RR indicated in bold type.

### 3.2 Perinatal Cd Exposure Induces IDA, Which Can Be Rescued by Fe Supplementation

**FIGURE 5:**
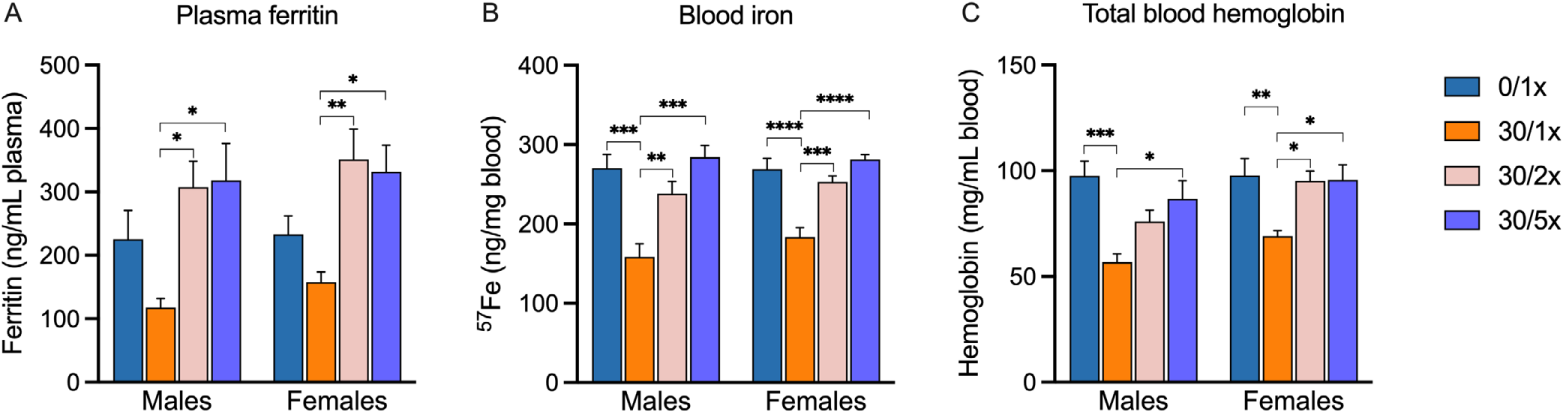
Iron status. (A) Plasma ferritin, (B) blood iron (^57^Fe), and (C) total blood hemoglobin levels. *p < 0.05; **p < 0.01; ***p < 0.001; and ****p < 0.0001.

To explore both the effect of developmental Cd exposure on Fe and IDA status, and the ability of Cd‐exposed mice to absorb Fe, we measured circulating ferritin (Figure 5A, File S1), elemental Fe (Figure 5B, File S1), and hemoglobin (Figure 5C, File S1) in PND21 offspring. Ferritin levels were not significantly reduced due to Cd alone, but both the 30/2× and 30/5× groups displayed significantly higher ferritin levels than the 30/1× group, suggesting Cd does not interfere with Fe storage. Cd alone significantly decreased the circulating elemental Fe in both male and female offspring, and adding 2× and 5× dietary Fe was sufficient to raise Fe levels in a dose‐responsive manner, without exceeding normal values. Other essential trace metals were also measured, and in males, manganese (Mn) levels were elevated in the 30/2× group only, and copper (Cu) was reduced in the 30/1× and 30/2× groups (Figure S4A, File S1). Levels of other essential trace metals were unaltered in females (Figure S4B, File S1). Cd levels were below the LOQ in > 85% of samples in each treatment group (Figure S4A,B, File S1). Hemoglobin levels decreased due to Cd and were fully rescued in both males and females by Fe supplementation, with the exception of partial rescue by 2× Fe in males, indicating that perinatal Cd induced not only ID but IDA in juvenile offspring.

**FIGURE 6:**
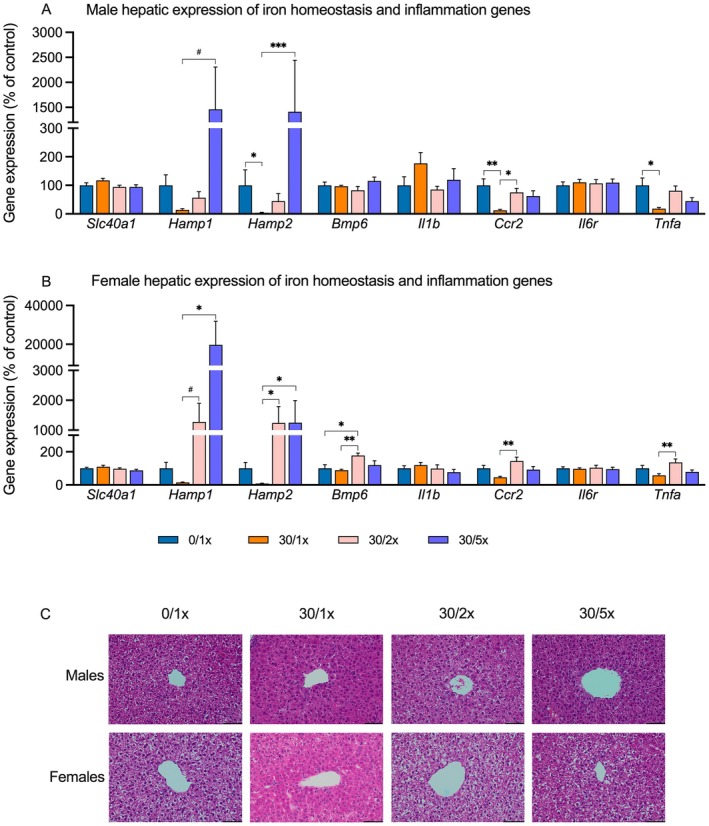
Hepatic iron homeostasis and inflammation. Hepatic expression of genes related to iron homeostasis and inflammation in males (A) and females (B). 40× photomicrographs (C) of hematoxylin and eosin (H&E) staining in hepatic tissue; scale bar indicates 100 μm length. *p < 0.05; **p < 0.01; ***p < 0.001; and ^#^ *p* < 0.1.

To determine the effect of Cd alone and in combination with Fe supplementation on Fe homeostasis in the liver, we performed qRT‐PCR on the following genes: *Slc40a1*, which encodes ferroportin, the protein responsible for Fe absorption, transport, and homeostasis; *Hamp1* and *Hamp2*, which encode hepcidin, the protein that prevents Fe overload; and *Bmp6*, which encodes a protein that regulates hepcidin (Figure 6A,B, File S1). There was no effect of either Cd alone or in combination with Fe supplementation on *Slc40a1*. In general, hepcidin gene expression trended downward due to Cd alone, though only significantly so in the case of male *Hamp2*. In males, the addition of 5× dietary Fe led to significant upregulation of *Hamp2* and non‐significant upregulation of *Hamp1* compared to the Cd only group. In females, both Fe supplementation groups exhibited hepcidin upregulation compared to the Cd only group, although not significantly so for *Hamp1* expression in the 30/2× group. *Bmp6* expression was unchanged in male offspring, but in females, the 30/2× group displayed upregulation compared to both the 0/1× and the 30/1× groups.

To establish whether the selected Fe supplementation dosages themselves caused inflammation or hepatic Fe overload, we performed qRT‐PCR to assess hepatic expression of the inflammation genes *Il1b*, *Il6r*, *Ccr2*, and *Tnfa* (Figure 6A,B, File S1), along with H&E histology to detect Fe deposits and general morphological features of inflammation. Neither males nor females exhibited dysregulation of *Il1b* or *Il6r*. In males, both *Ccr2* expression and *Tnfa* expression were significantly downregulated in the 30/1× group compared to the 0/1× group, with partial or full rescue by Fe supplementation. In females, *Ccr2* expression and *Tnfa* expression were downregulated in the 30/1× group only when compared to the 30/2× group. There was no dysregulation of any inflammation gene in response to Fe supplementation compared to the control group. Representative liver samples were inspected for Fe deposits indicative of Fe overload, and none were found in any group (Figure 6C, File S1).

### 3.3 Fe Supplementation Is Sufficient to Prevent Perinatal Cd‐Induced Juvenile MASLD

**FIGURE 7:**
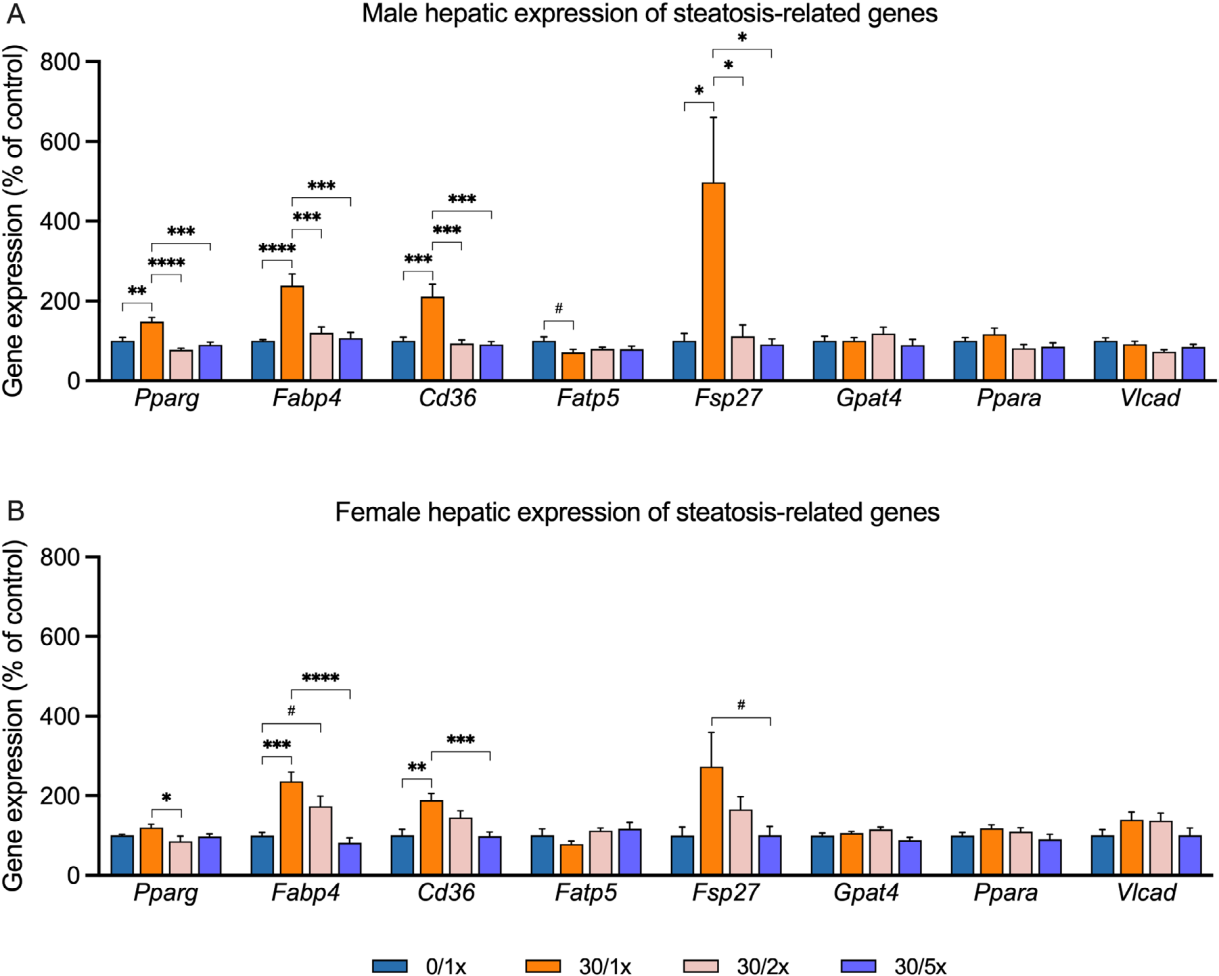
Hepatic expression of genes related to lipid homeostasis and steatosis in males (A) and females (B). *p < 0.05; **p < 0.01; ***p < 0.001; ****p < 0.0001, and ^#^ *p* < 0.1.

We have previously demonstrated that perinatal Cd exposure induces hepatic steatosis, characterized by upregulation of lipid homeostasis genes and hepatic lipid deposition, and a hallmark of MASLD. To investigate a role for Fe deficiency in perinatal Cd‐induced steatosis, we used qRT‐PCR to evaluate the impact of perinatal Cd exposure with and without Fe supplementation on hepatic transcription of genes related to fatty acid transport (*Pparg, Fabp4, Cd36*, and *Fatp5*), TAG accumulation (*Fsp27* and *Gpat4*), and β‐oxidation (*Ppara* and *Vlcad*). Males exhibited significant Cd‐induced upregulation of three out of four fatty acid import genes, *Pparg*, *Fabp4*, and *Cd36*, all of which were completely rescued to normal levels with either 2× or 5× Fe supplementation (Figure 7A, File S1). The remaining fatty acid import gene, *Fatp5*, exhibited a non‐significant decrease in expression due to Cd alone that was not present in either Fe supplementation group. *Fsp27*, which encodes a protein that reduces fatty acid oxidation and contributes to hepatic TAG accumulation, was significantly upregulated by Cd alone compared to all other groups. There was no effect of Cd or Fe on hepatic expression of *Gpat4, Ppara*, or *Vlcad*. In general, similar gene expression trends were displayed in females, although with less robust intergroup differences (Figure 7B, File S1). *Pparg* expression was significantly increased due to Cd alone compared to the 30/2× group, and *Fabp4* and *Cd36* were both upregulated by Cd alone and returned to normal expression levels in the 30/5× group. There were no significant effects of Cd or Fe on expression of *Fatp5, Fsp27, Gpat4, Ppara*, or *Vlcad* in females, though changes in *Fsp27* followed similar trends to those seen in male offspring.

**FIGURE 8:**
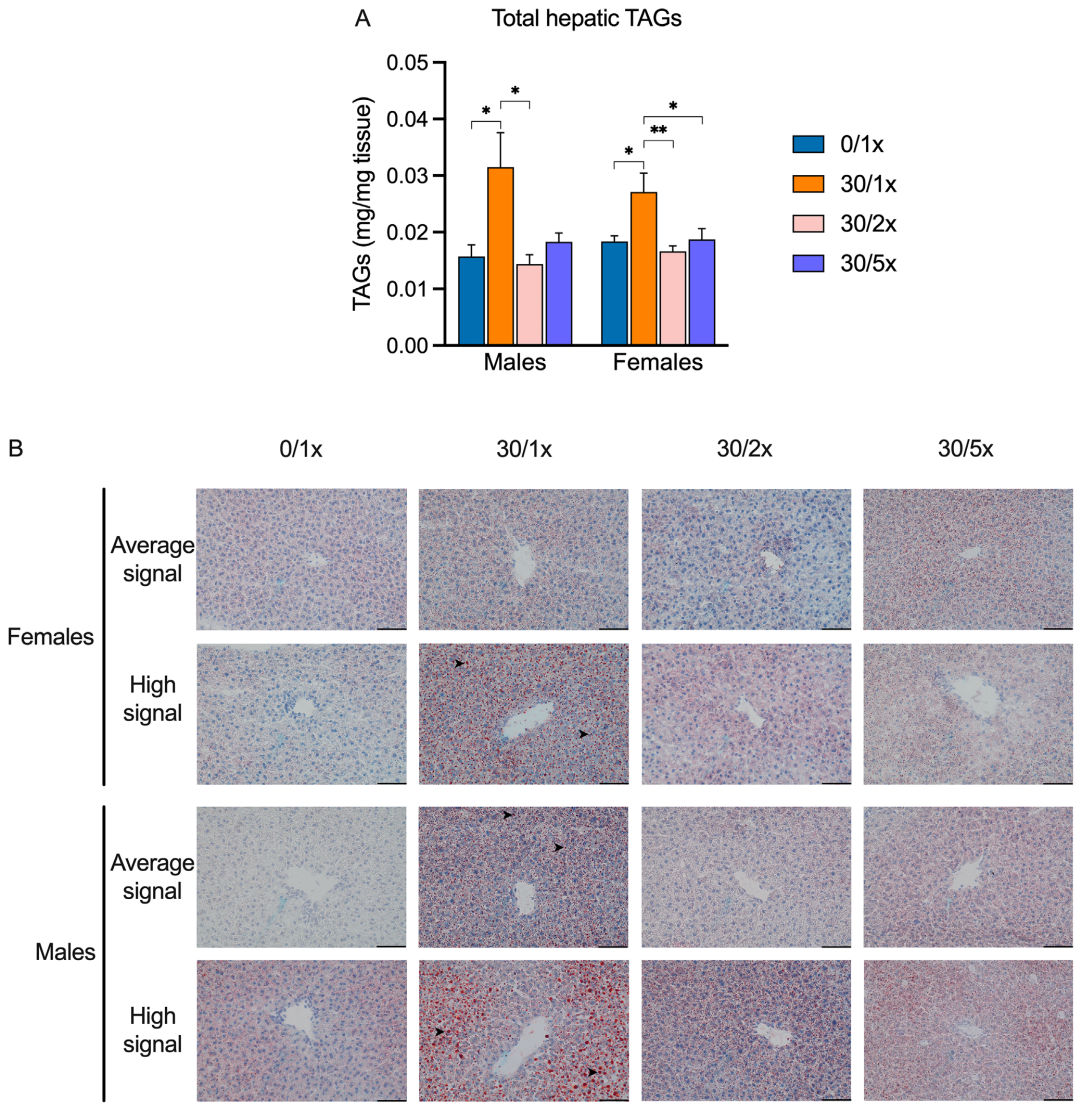
Hepatic steatosis. (A) Total hepatic triacylglyceride (TAG) abundance. (B) 40× photomicrographs of Oil Red O (ORO) histochemistry in hepatic tissue samples displaying average and high TAG abundance; scale bar indicates 100 μm length. Neutral lipid deposits are stained in red; representative droplets are indicated with arrowheads. *p < 0.05; **p < 0.01.

To determine if changes in programming of lipid homeostasis gene expression had an impact on lipid deposition, we quantified total hepatic TAGs directly (Figure 8A, File S1) and visualized neutral lipid droplets by ORO staining of tissue sections (Figure 8B, File S1). Consistent with previous findings, a greater abundance of TAGs was detected in the liver of male and female offspring in the 30/1× group. In males and females, TAG levels return to normal in the 30/2× group. Females also displayed full recovery in the 30/5× group, while males exhibited partial recovery. We then selected liver samples for ORO histochemistry that represented those with the highest TAG abundance and those closest to the mean TAG abundance in each group. We detected remarkable ORO signal in the high TAG male, average TAG female, and high TAG female samples from the 30/1× group, while samples from the Fe supplemented groups appeared similar to the controls.

**FIGURE 9:**
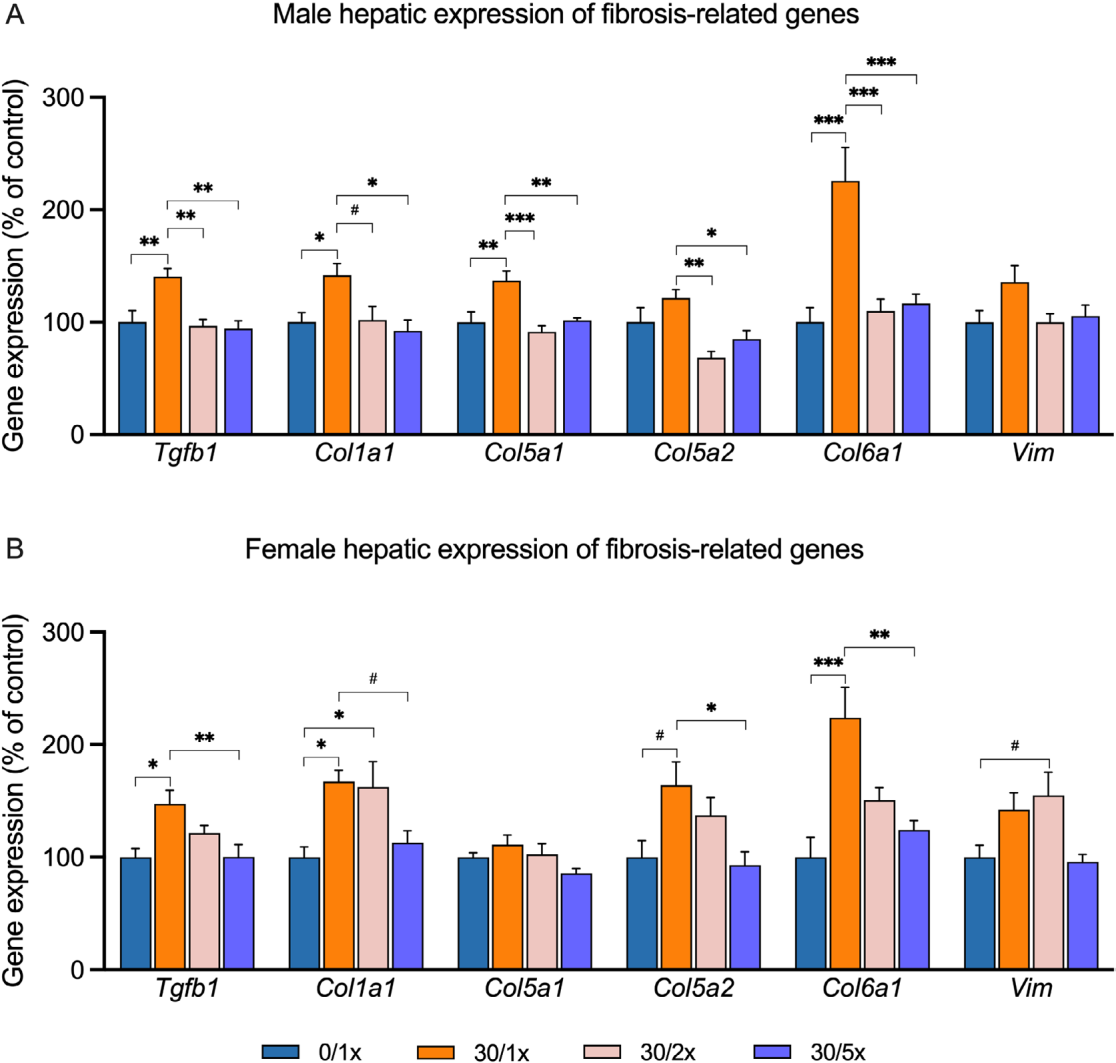
Hepatic expression of genes related to fibrosis in (A) males and (B) females. *p < 0.05; **p < 0.01; ***p < 0.001; and ^#^ *p* < 0.1.

Another feature of perinatal Cd‐induced juvenile MASLD that we previously documented is hepatic fibrosis. To determine the impact of Fe supplementation on perinatal Cd‐induced profibrogenic programming, we performed qRT‐PCR for gene expression of *Tgfb1*, a chief promotor of extracellular matrix (ECM) production; the procollagen isoforms *Col1a1, Col5a1, Col5a2*, and *Col6a1*; and *Vim*, a signature of activated hepatic stellate cells, in both male (Figure 9A, File S1) and female (Figure 9B, File S1) offspring. In males, expression of *Tgfb1, Col1a1, Col5a1*, and *Col6a1* was activated by Cd alone and rescued by Fe supplementation. Expression of *Col5a2* was significantly higher in the Cd only group compared to both Fe‐treated groups. There was no effect of Cd alone or with Fe on *Vim* expression. In females, *Tgfb1* and *Col6a1* expression was activated by perinatal Cd in the 30/1× group and the 30/2× group but was partially rescued by 5× Fe. *Col5a2* expression trended toward upregulation in the 30/1× group compared to the 0/1× group and significantly so compared to the 30/5× group. Expression of *Vim* trended toward upregulation in the 30/2× group compared to controls, and there were no significant intergroup differences in *Col5a2* expression.

**FIGURE 10:**
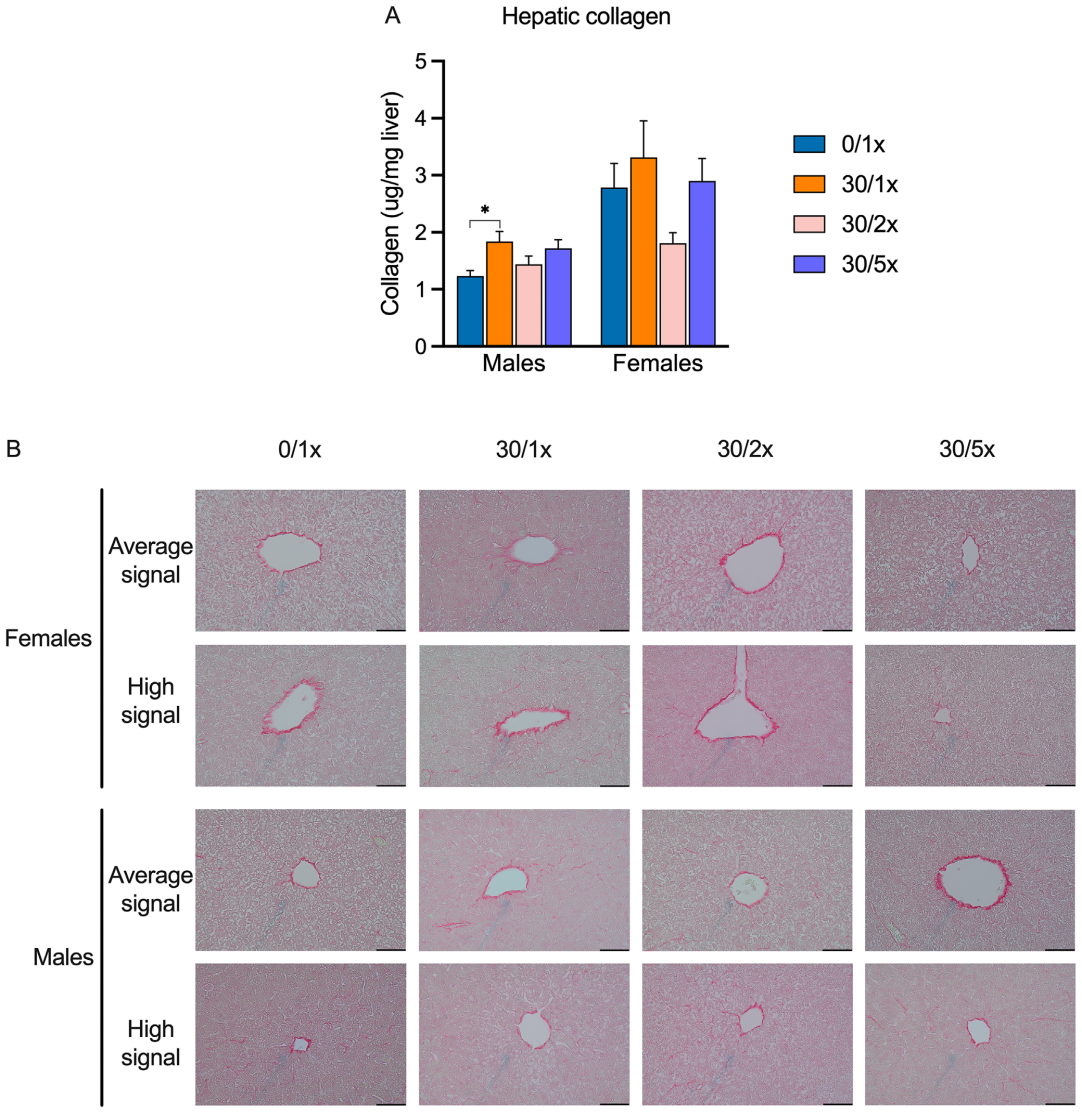
Hepatic fibrosis. (A) Total hepatic collagen abundance. (B) 40× photomicrographs of Sirius Red (SR) histochemistry in hepatic tissue samples displaying average and high collagen abundance; scale bar indicates 100 μm. Collagen fibers are stained red. *p < 0.05.

To determine how changes in expression of ECM and fibrosis programming genes related to hepatic collagen levels, we performed a hydroxyproline detection assay to quantify total hepatic collagen (Figure 10A, File S1) and Sirius red (SR) staining of hepatic tissue to visualize collagen infiltration (Figure 10B, File S1). Male offspring exposed to Cd alone had significantly more hepatic collagen, but Fe supplementation only led to partial rescue. In females, there were no significant intergroup differences. Imaging of SR‐stained hepatic sections revealed only the expected collagen fibers surrounding the portal veins, and no remarkable fibrosis in any group, male or female.

**FIGURE 11:**
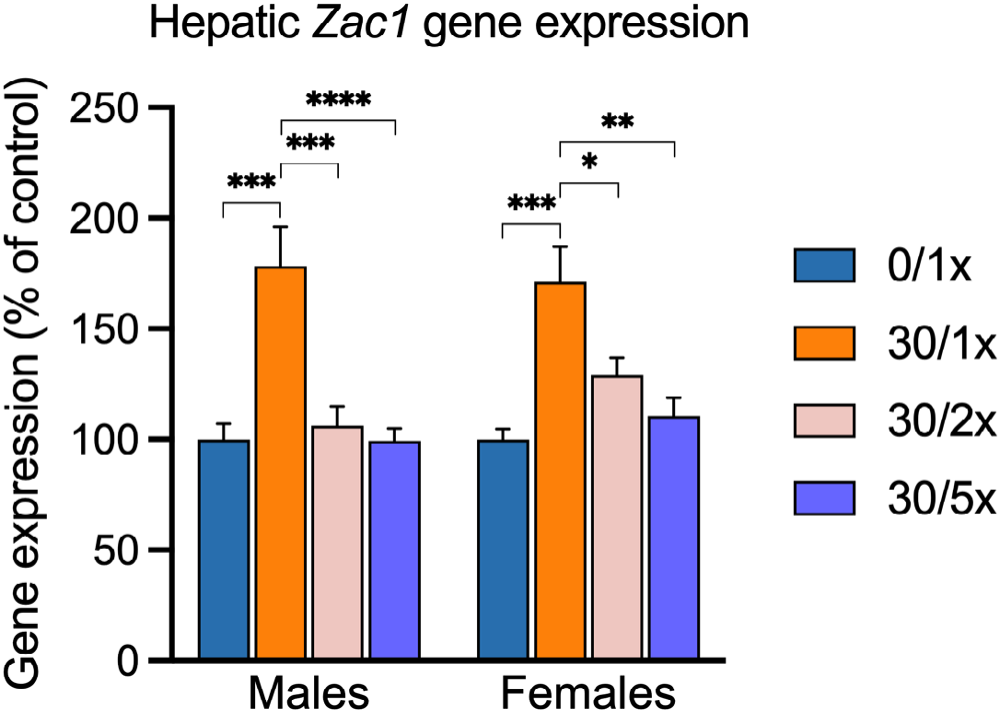
Hepatic gene expression of *Zac1*. *p < 0.05; **p < 0.01; ***p < 0.001; and ****p < 0.0001.

We previously determined that the imprinted gene *Zac1* drives hepatic steatosis and fibrosis in our model of perinatal Cd exposure [31]. Consistent with our previous findings, *Zac1* expression was significantly upregulated in the liver in response to Cd alone in both males and females in the present study (Figure 11, File S1). This increased expression was completely rescued by 2× and 5× Fe supplementation, adding further weight to a role for Fe deficiency in perinatal Cd‐induced juvenile MASLD.

## 4 Discussion

ID and IDA are highly prevalent conditions, especially during pregnancy. Although these conditions are often linked to dietary deficiencies, genetic predisposition, or the physiological demands of the gestating fetus, exposure to toxic metals is frequently overlooked as a contributor. Nearly half a century ago, researchers first experimentally demonstrated that both maternal Cd exposure and maternal ID led to similar fetal presentations with FGR [54]. Since then, numerous studies have linked Cd exposure to ID and IDA [9, 12, 13, 18, 19, 20, 21, 55, 56]. We have previously shown that our model of generating maternal Cd burden renders offspring Fe‐deficient at birth [12], and our findings reported here provide further support for this postulate and demonstrate that the phenomenon persists until PND21. Under normal circumstances, the majority of Fe is bound to hemoglobin, a protein responsible for transporting oxygen to every organ, and the rest is bound to ferritin, a protein complex that sequesters Fe for storage [57, 58]. Here we showed that hemoglobin levels are lower in Cd‐exposed offspring, indicating they are not only suffering from ID but that it has progressed to IDA. Surprisingly, circulating ferritin was not significantly lower due to Cd. Together, this pattern suggests that stored Fe was somewhat buffered from the effects of Cd, but that the overall elemental Fe deficiency was largely due to the hemoglobin deficit. One of the key goals of this study was to determine whether Fe supplementation could have any effect on Cd‐induced iron deficiency due to evidence from the literature that Cd exposure blocks Fe absorption [9, 10, 11]. As part of the earliest studies that linked prenatal Cd, ID, and FGR, Fe injection was shown to be partially effective at resolving Cd‐induced fetal ID [54, 59]. In the presently reported study, we are the first to investigate whether dietary Fe supplementation can alleviate Cd‐induced ID and IDA in offspring at a later timepoint, PND21. When treated with a diet containing 1060 ppm ferric citrate, 5× the amount contained in the standard AIN‐93G, both elemental Fe and hemoglobin levels return to the normal levels seen in control offspring. 2× Fe supplementation was sufficient to significantly raise elemental Fe levels; we did observe that absolute mean values remained below those of controls, though not significantly so. Our findings support the idea that perinatal Cd exposure causes IDA in offspring, despite access to a nutrient sufficient diet, and that Cd‐induced IDA at PND21 can be prevented by dietary Fe fortification.

To determine the effects of Fe supplementation combined with Cd exposure on Fe homeostasis and to ensure that fortifying the diet with excess Fe did not lead to Fe overload, we examined the expression of several genes responsible for controlling Fe levels related to inflammation in offspring liver. The most notable effect was on the two genes that encode for hepcidin, a protein responsible for preventing Fe overload. Fe supplementation tended to promote transcription of *Hamp1* and *Hamp2*, though there was considerable variability in this response. Hepcidin upregulation, along with upregulation of *Bmp6*, a gene that encodes for a hepcidin regulator, suggests that the proper compensatory response to excess Fe was undertaken. Cd led to downregulation of *Ccr2*, an outcome that typically occurs as monocytes differentiate into macrophages as part of the immune response [60]. Surprisingly, *Tnfa* expression was also downregulated in males, suggesting Cd exposure decreased the hepatic immune response, which could leave offspring vulnerable to infection [61]. Any Cd‐induced changes to the inflammatory response were not exacerbated by supplementary Fe, suggesting the chosen fortification levels did not induce hepatic Fe toxicity. In fact, iron deposits were not detected upon visual inspection of H&E‐stained liver sections.

Fe supplementation was also effective at returning perinatal Cd‐induced changes to body weight, organ weights, and hair growth patterns to normal conditions. Growth restriction is one of the most recognizable consequences of early life Cd exposure [12, 31, 62, 63, 64, 65]. The brain sparing effect, cardiac hypertrophy, and liver atrophy have also been documented [12, 31, 46]. Growth restriction and cardiac hypertrophy have also been independently linked to IDA, outside of the context of Cd exposure [66, 67, 68, 69]. The growth restriction phenotype was more significant and more completely rescued in males, a pattern with precedent [70], although others have found that female offspring are more sensitive [71, 72]. The brain sparing effect was only present in males, and it was not fully rescued by Fe, emphasizing the strength of the drive to preserve brain size in the face of toxicant assault. Cd‐induced cardiomegaly and dose‐responsive rescue by supplemental Fe are robustly exhibited in both male and female offspring. The mechanism underlying the association between IDA and cardiomegaly has not been conclusively determined, but a leading theory is that the heart muscle becomes hypertrophic to compensate for the inadequate hemoglobin load to deliver sufficient oxygen to maintain organ function [73].

Finally, we attempted to determine if concurrent Fe supplementation could prevent perinatal Cd‐induced juvenile MASLD. We previously showed that Cd alone, without changing dietary composition, caused offspring to present with liver steatosis and fibrosis at PND21 [31]. Here we report that perinatal Cd programmed the upregulation of hepatic genes related to steatosis and fibrosis, caused overt steatosis in males and females, and fibrosis in males. In nearly every case, Fe supplementation prevented the development of these MASLD‐related outcomes. The most striking effects were found in male offspring. Genes that regulate fatty acid import and lipid droplet deposition were all upregulated by Cd alone and fully returned to normal expression levels when Fe fortification was provided. These molecular changes were mirrored phenotypically, as indicated by levels of hepatic TAGs. Fe supplementation also partially prevented hepatic fibrosis in males. Females, overall, appeared less sensitive to Cd, as fewer MASLD genes were dysregulated, and there was no evidence of fibrosis. Interestingly, females also appeared less responsive to the protective effects of Fe; specifically, a higher Fe dosage was required to rescue the expression of most fibrosis genes in females, while the lower dose was sufficient in males. The exact mechanism by which Fe supplementation prevents MASLD remains unclear, but several theories have been proposed. MASLD and IDA can be concomitant. Chronic liver inflammation causes hepcidin upregulation, which hinders Fe absorption and bioavailability [42, 43]. However, this postulate is not consistent with our findings; maternal Cd exposure reduces hepcidin and does not elicit a strong inflammatory response. Others have proposed a model in which fatty acid metabolism flags in the absence of Fe, a crucial cofactor of mitochondrial respiration [42, 43]. Maternal Cd exposure has been linked to hepatic mitochondrial dysfunction, so this avenue presents a promising research direction.

Our findings highlight the potential for oral Fe supplementation as a therapeutic intervention to prevent growth restriction and juvenile MASLD in the offspring of Cd‐exposed mothers. Furthermore, our finding that ferritin levels may not be affected before the progression to profound IDA promotes the need for heavy metal screening before and during pregnancy. For example, a patient may not present as Fe‐deficient from the typical ferritin screening test but may still benefit from Fe supplementation if they are carrying a high Cd burden. Cd levels are not routinely measured during pregnancy unless there is a specific reason for suspected exposure because, unlike those for lead, the American College of Obstetricians and Gynecologists does not provide Cd screening guidelines [74]. We have also demonstrated that even relatively low‐level Fe fortification, within the recommended dosage of Fe during an anemic pregnancy, can remedy the effects of Cd on both elemental Fe and hemoglobin levels, in contrast to previous studies showing that Cd damaged Fe absorption machinery, implying excess Fe would have no effect. Our findings related to MASLD are especially intriguing, as an early study found that maternal Fe supplementation partially rescued FGR but did not investigate liver health [59]. We acknowledge that this study has several limitations, including the unknown implications of our findings as the mouse ages past PND21. There is a challenge in extrapolating results from a mouse model to human health; although we did not observe overt Fe toxicity in our model, further studies must be undertaken to ensure that Fe supplementation levels translate effectively. Additionally, although Fe is clearly implicated in the developmental toxicity of Cd, the exact mechanism that underlies this connection has not been determined. Because there was no detected Cd in any group, we cannot conclude that Fe supplementation affected Cd absorption in pups. Further studies are planned that will investigate the dynamics of Cd and Fe absorption in the maternal generation and the developing fetus.

## Author Contributions

Rebecca Lichtler and Michael Cowley designed the experiments. Rebecca Lichtler, Hannah Klossner, and Nikia Smith performed the experiments, analyzed the data, and generated the figures. Michael Cowley and Cathrine Hoyo provided support and funding for the experiments. Rebecca Lichtler and Michael Cowley wrote and edited the manuscript.

## Conflicts of Interest

The authors declare no conflicts of interest.

## Supporting information


Data S1.

Data S2.

Table S1.

Table S2.

## Data Availability

The data that support the findings of this study are available in the Materials and Methods, Results, and/or Supporting Information of this article.

## References

[1] A. Kubier , R. T. Wilkin , and T. Pichler , “Cadmium in Soils and Groundwater: A Review,” Applied Geochemistry 108 (2019): 1–16, 10.1016/j.apgeochem.2019.104388. PMC7147761 32280158

[2] G. Scherer and H. Barkemeyer , “Cadmium Concentrations in Tobacco and Tobacco Smoke,” Ecotoxicology and Environmental Safety 7 (1983): 71–78, 10.1016/0147-6513(83)90050-7. 6851927

[3] K. Wróblewski , J. Wojnicka , P. Tutka , A. Szmagara , and A. Błażewicz , “Measurements of Cadmium Levels in Relation to Tobacco Dependence and as a Function of Cytisine Administration,” Scientific Reports 14 (2024): 1883, 10.1038/s41598-024-52234-w. 38253706 PMC10803351

[4] A. Turner , “Cadmium Pigments in Consumer Products and Their Health Risks,” Science of the Total Environment 657 (2019): 1409–1418, 10.1016/j.scitotenv.2018.12.096. 30677907

[5] WHO , “Exposure to Cadmium: A Major Public Health Concern,” (2019), https://www.who.int/publications/i/item/WHO‐CED‐PHE‐EPE‐19‐4‐3.

[6] T. Leazer , “Cadmium Absorption and Its Relationship to Divalent Metal Transporter‐1 in the Pregnant Rat,” Toxicology and Applied Pharmacology 185 (2002): 18–24, 10.1006/taap.2002.9505. 12460733

[7] A. Espart , S. Artime , G. Tort‐Nasarre , and E. Yara‐Varón , “Cadmium Exposure During Pregnancy and Lactation: Materno‐Fetal and Newborn Repercussions of Cd(II), and Cd–Metallothionein Complexes,” Metallomics 10 (2018): 1359–1367, 10.1039/c8mt00174j. 30221266

[8] G. Genchi , M. S. Sinicropi , G. Lauria , A. Carocci , and A. Catalano , “The Effects of Cadmium Toxicity,” International Journal of Environmental Research and Public Health 17 (2020): 3782, 10.3390/IJERPH17113782. 32466586 PMC7312803

[9] M. Tokumoto , J.‐Y. Lee , Y. Fujiwara , and M. Satoh , “Long‐Term Exposure to Cadmium Causes Hepatic Iron Deficiency Through the Suppression of Iron‐Transport‐Related Gene Expression in the Proximal Duodenum,” Toxics 11 (2023): 641, 10.3390/toxics11070641. 37505606 PMC10386400

[10] Y. Fujiwara , J.‐Y. Lee , H. Banno , et al., “Cadmium Induces Iron Deficiency Anemia Through the Suppression of Iron Transport in the Duodenum,” Toxicology Letters 332 (2020): 130–139, 10.1016/j.toxlet.2020.07.005. 32645461

[11] H. Horiguchi , E. Oguma , and F. Kayama , “Cadmium Induces Anemia Through Interdependent Progress of Hemolysis, Body Iron Accumulation, and Insufficient Erythropoietin Production in Rats,” Toxicological Sciences 122 (2011): 198–210, 10.1093/toxsci/kfr100. 21540277

[12] K. M. Hudson , S. M. Belcher , and M. Cowley , “Maternal Cadmium Exposure in the Mouse Leads to Increased Heart Weight at Birth and Programs Susceptibility to Hypertension in Adulthood,” Scientific Reports 9 (2019): 13553, 10.1038/s41598-019-49807-5. 31537853 PMC6753073

[13] K. Zhang , M. Long , W. Dong , et al., “Cadmium Induces Kidney Iron Deficiency and Chronic Kidney Injury by Interfering With the Iron Metabolism in Rats,” International Journal of Molecular Sciences 25 (2024): 763, 10.3390/ijms25020763. 38255838 PMC10815742

[14] B. C. Kennedy , D. J. Wallin , P. V. Tran , and M. K. Georgieff , “Long‐Term Brain and Behavioral Consequences of Early‐Life Iron Deficiency,” in Fetal Development (Springer International Publishing, 2016), 295–316, 10.1007/978-3-319-22023-9_15.

[15] M. K. Georgieff , “Long‐Term Brain and Behavioral Consequences of Early Iron Deficiency,” Nutrition Reviews 69, no. Suppl 1 (2011): S43–S48, 10.1111/j.1753-4887.2011.00432.x. 22043882 PMC3266848

[16] N. M. Abu‐Ouf and M. M. Jan , “The Impact of Maternal Iron Deficiency and Iron Deficiency Anemia on Child's Health,” Saudi Medical Journal 36 (2015): 146–149, 10.15537/smj.2015.2.10289. 25719576 PMC4375689

[17] M. A. Kharate and S. G. Choudhari , “Effects of Maternal Anemia Affecting Fetal Outcomes: A Narrative Review,” Cureus 16 (2024): e64800, 10.7759/cureus.64800. 39156476 PMC11330297

[18] M. Ghoochani , M. H. Dehghani , N. Rastkari , et al., “Association Among Sources Exposure of Cadmium in the Adult Non‐Smoking General Population of Tehran,” Biological Trace Element Research 191 (2019): 27–33, 10.1007/s12011-018-1590-9. 30535673

[19] Y. Kim , D. T. Lobdell , C. W. Wright , V. V. Gocheva , E. Hudgens , and R. M. Bowler , “Blood Metal Concentrations of Manganese, Lead, and Cadmium in Relation to Serum Ferritin Levels in Ohio Residents,” Biological Trace Element Research 165 (2015): 1–9, 10.1007/s12011-014-0223-1. 25578336

[20] C. M. Gallagher , J. J. Chen , and J. S. Kovach , “The Relationship Between Body Iron Stores and Blood and Urine Cadmium Concentrations in US Never‐Smoking, Non‐Pregnant Women Aged 20‐49 Years,” Environmental Research 111 (2011): 702–707, 10.1016/j.envres.2011.03.007. 21507392

[21] E. Örün , S. S. Yalçın , and O. Aykut , “Lead, Mercury, and Cadmium Levels in Breast Milk and Infant Hair in the Late Period of Lactation in Ankara, Turkey,” International Journal of Environmental Health Research 32 (2022): 1950–1961, 10.1080/09603123.2021.1929872. 34092151

[22] R. Lichtler and M. Cowley , “Environmental Contaminants, Iron Deficiency, and Iron Deficiency‐Anemia: A Review of the Literature,” Scientifica (2025). 10.1155/sci5/5007983 PMC12436012 40959526

[23] S.‐J. Yi , Y.‐W. Xiong , H.‐L. Zhu , et al., “Environmental Cadmium Exposure During Pregnancy Causes Diabetes‐Like Phenotypes in Mouse Offspring: Association With Oxidative Stress in the Fetal Liver,” Science of the Total Environment 777 (2021): 146006, 10.1016/j.scitotenv.2021.146006. 33677283

[24] M. Yoruk , M. Kanter , I. Meral , and Z. Agaoglu , “Localization of Glycogen in the Placenta and Fetal and Maternal Livers of Cadmium‐Exposed Diabetic Pregnant Rats,” Biological Trace Element Research 96 (2003): 217–226, 10.1385/BTER:96:1-3:217. 14716101

[25] P. Xu , J. Guo , Y. Jin , et al., “Toxic Effects of Maternal Cadmium Exposure on the Metabolism and Transport System of Amino Acids in the Maternal Livers,” Ecotoxicology and Environmental Safety 254 (2023): 114726, 10.1016/J.ECOENV.2023.114726. 36898312

[26] O. Hyder , M. Chung , D. Cosgrove , et al., “Cadmium Exposure and Liver Disease Among US Adults,” Journal of Gastrointestinal Surgery 17 (2013): 1265–1273, 10.1007/s11605-013-2210-9. 23636881 PMC3974907

[27] S. Bellentani , F. Scaglioni , M. Marino , and G. Bedogni , “Epidemiology of Non‐Alcoholic Fatty Liver Disease,” Digestive Diseases 28 (2010): 155–161, 10.1159/000282080. 20460905

[28] A. Koyu , A. Gokcimen , F. Ozguner , D. S. Bayram , and A. Kocak , “Evaluation of the Effects of Cadmium on Rat Liver,” Molecular and Cellular Biochemistry 284 (2006): 81–85, 10.1007/s11010-005-9017-2. 16424996

[29] H. Kurita , H. Nagase , M. Tokumoto , J.‐Y. Lee , and M. Satoh , “DNA Microarray Analysis of Fetal Liver of C57BL/6J Mice Exposed to Cadmium During Gestation,” Fundamental Toxicological Sciences 3 (2016): 257–280, 10.2131/fts.3.257.

[30] Y. T. Fu , J. Zhang , W. B. Liu , et al., “Gestational Cadmium Exposure Disrupts Fetal Liver Development via Repressing Estrogen Biosynthesis in Placental Trophoblasts,” Food and Chemical Toxicology 176 (2023): 113807, 10.1016/J.FCT.2023.113807. 37121429

[31] S. D. Riegl , C. Starnes , D. D. Jima , et al., “The Imprinted Gene *Zac1* Regulates Steatosis in Developmental Cadmium‐Induced Nonalcoholic Fatty Liver Disease,” Toxicological Sciences 191 (2023): 34–46, 10.1093/toxsci/kfac106. 36200916 PMC9887675

[32] J. Kuriwaki , M. Nishijo , R. Honda , et al., “Effects of Cadmium Exposure During Pregnancy on Trace Elements in Fetal Rat Liver and Kidney,” Toxicology Letters 156 (2005): 369–376, 10.1016/j.toxlet.2004.12.009. 15763636

[33] S. Satarug , “Long‐Term Exposure to Cadmium in Food and Cigarette Smoke, Liver Effects and Hepatocellular Carcinoma,” Current Drug Metabolism 13 (2012): 257–271, 10.2174/138920012799320446. 22455552

[34] Y. M. Go , R. L. Sutliff , J. D. Chandler , et al., “Low‐Dose Cadmium Causes Metabolic and Genetic Dysregulation Associated With Fatty Liver Disease in Mice,” Toxicological Sciences 147 (2015): 524–534, 10.1093/toxsci/kfv149. 26187450 PMC4598795

[35] H. Men , J. L. Young , W. Zhou , et al., “Early‐Life Exposure to Low‐Dose Cadmium Accelerates Diethylnitrosamine and Diet‐Induced Liver Cancer,” Oxidative Medicine and Cellular Longevity 2021 (2021): 1427787, 10.1155/2021/1427787. 34876963 PMC8645401

[36] D. Hong , J. Y. Min , and K. B. Min , “Association Between Cadmium Exposure and Liver Function in Adults in the United States: A Cross‐Sectional Study,” Journal of Preventive Medicine and Public Health 54 (2021): 471–480, 10.3961/jpmph.21.435. 34875830 PMC8655368

[37] H. Kurita , T. Hasegawa , Y. Seko , et al., “Effect of Gestational Cadmium Exposure on Fetal Growth, Polyubiquitinated Protein and Monoubiqutin Levels in the Fetal Liver of Mice,” Journal of Toxicological Sciences 43 (2018): 19–24, 10.2131/jts.43.19. 29415948

[38] C. Liu , Y. Zhu , Z. Lu , et al., “Cadmium Induces Acute Liver Injury by Inhibiting Nrf2 and the Role of NF‐κB, NLRP3, and MAPKS Signaling Pathway,” International Journal of Environmental Research and Public Health 17 (2020): 138, 10.3390/ijerph17010138. PMC6981660 31878134

[39] W. Hazelhoff Roelfzema , A. M. Roelofsen , J. H. Copius Peereboom‐Stegeman , and C. J. Van Noorden , “Glycogen Content of Placenta and of Fetal and Maternal Liver of Cadmium‐Exposed Rats. II: A Quantitative Histochemical Study,” Placenta 9 (1988): 39–45, 10.1016/0143-4004(88)90071-9. 3362792

[40] W.‐K. Chan , K.‐H. Chuah , R. B. Rajaram , L.‐L. Lim , J. Ratnasingam , and S. R. Vethakkan , “Metabolic Dysfunction‐Associated Steatotic Liver Disease (MASLD): A State‐of‐the‐Art Review,” Journal of Obesity & Metabolic Syndrome 32 (2023): 197–213, 10.7570/jomes23052. 37700494 PMC10583766

[41] A. A. Parsa , K. A. Azama , M. Vawer , M. A. Ona , and T. B. Seto , “Prevalence Study of MASLD in Adolescent and Young Adult Pacific Islanders and Asians Living in Hawai'i,” Journal of the Endocrine Society 8 (2024): bvad165, 10.1210/jendso/bvad165. 38249431 PMC10797323

[42] A. Siddique , J. E. Nelson , B. Aouizerat , M. M. Yeh , K. V. Kowdley , and NASH Clinical Research Network , “Iron Deficiency in Patients With Nonalcoholic Fatty Liver Disease Is Associated With Obesity, Female Gender, and Low Serum Hepcidin,” Clinical Gastroenterology and Hepatology 12 (2014): 1170–1178, 10.1016/j.cgh.2013.11.017. 24269922 PMC4028425

[43] H.‐H. Yang , G.‐C. Chen , D.‐M. Li , et al., “Serum Iron and Risk of Nonalcoholic Fatty Liver Disease and Advanced Hepatic Fibrosis in US Adults,” Scientific Reports 11 (2021): 10387, 10.1038/s41598-021-89991-x. 34002001 PMC8128903

[44] T. Mitchell , E. McKinnon , O. Ayonrinde , et al., “Decreased Physical Working Capacity in Adolescents With Nonalcoholic Fatty Liver Disease Associates With Reduced Iron Availability,” Clinical Gastroenterology and Hepatology 18 (2020): 1584–1591, 10.1016/j.cgh.2019.10.017. 31628998

[45] M. D. Simmers , K. M. Hudson , M. Baptissart , and M. Cowley , “Epigenetic Control of the Imprinted Growth Regulator *Cdkn1c* in Cadmium‐Induced Placental Dysfunction,” Epigenetics 18, no. 1 (2023): 2088173, 10.1080/15592294.2022.2088173. 35770551 PMC10989690

[46] K. M. Hudson , E. Shiver , J. Yu , et al., “Transcriptomic, Proteomic, and Metabolomic Analyses Identify Candidate Pathways Linking Maternal Cadmium Exposure to Altered Neurodevelopment and Behavior,” Scientific Reports 11 (2021): 16302, 10.1038/s41598-021-95630-2. 34381081 PMC8357970

[47] K. K. Helfrich , N. Saini , S. T. C. Kwan , O. C. Rivera , R. Hodges , and S. M. Smith , “Gestational Iron Supplementation Improves Fetal Outcomes in a Rat Model of Prenatal Alcohol Exposure,” Nutrients 14 (2022): 1653, 10.3390/nu14081653. 35458215 PMC9025692

[48] S. T. C. Kwan , C. A. Kezer , K. K. Helfrich , et al., “Maternal Iron Nutriture Modulates Placental Development in a Rat Model of Fetal Alcohol Spectrum Disorder,” Alcohol 84 (2020): 57–66, 10.1016/j.alcohol.2019.11.003. 31734307 PMC7131893

[49] J. K. Lee , J.‐H. Ha , and J. F. Collins , “Dietary Iron Intake in Excess of Requirements Impairs Intestinal Copper Absorption in Sprague Dawley Rat Dams, Causing Copper Deficiency in Suckling Pups,” Biomedicine 9 (2021): 338, 10.3390/biomedicines9040338. PMC8065423 33801587

[50] K. J. Livak and T. D. Schmittgen , “Analysis of Relative Gene Expression Data Using Real‐Time Quantitative PCR and the 2^−ΔΔCT^ Method,” Methods 25 (2001): 402–408, 10.1006/meth.2001.1262. 11846609

[51] A. Mehlem , C. E. Hagberg , L. Muhl , U. Eriksson , and A. Falkevall , “Imaging of Neutral Lipids by Oil Red O for Analyzing the Metabolic Status in Health and Disease,” Nature Protocols 8 (2013): 1149–1154, 10.1038/nprot.2013.055. 23702831

[52] R. Lattouf , R. Younes , D. Lutomski , et al., “Picrosirius Red Staining,” Journal of Histochemistry and Cytochemistry 62 (2014): 751–758, 10.1369/0022155414545787. 25023614

[53] A. P. Shah , P. T. Patel , B. P. Patel , K. K. Mishra , and K. Ghosh , “Evaluation of Microtitre Plate‐Based Haemoglobin Estimation,” International Journal of Laboratory Hematology 40 (2018): 196–200, 10.1111/ijlh.12764. 29160616

[54] W. S. Webster , “Cadmium‐Induced Fetal Growth Retardation in the Mouse,” Archives of Environmental Health: An International Journal 33 (1978): 36–42, 10.1080/00039896.1978.10667306. 564669

[55] Q. Wang , J. Li , W. Ma , et al., “Blocked Erythroid Differentiation and Delayed Enucleation of Erythroblasts May Contribute to Murine Embryonic Toxicity Upon Exposure to Low Dose of Cadmium,” Toxicology Letters 387 (2023): 28–34, 10.1016/j.toxlet.2023.09.009. 37739093

[56] M. Wang , X. Wang , W. Cui , et al., “The Association Between Hemoglobin Level and Osteoporosis in a Chinese Population With Environmental Lead and Cadmium Exposure,” Environmental Geochemistry and Health 44 (2022): 1673–1682, 10.1007/s10653-021-01129-0. 34698982

[57] P. J. Aggett , “Iron,” in Present Knowledge in Nutrition, 10th ed., ed. J. W. Erdman , I. A. MacDonald , and S. H. Zeisel (Wiley, 2012), 506–520, 10.1002/9781119946045.ch33.

[58] C. A. Ross , B. Caballero , R. J. Cousins , and K. L. Tucker , Modern Nutrition in Health and Disease, 11th ed. (Lippincott Williams & Wilkins, 2014).

[59] W. S. Webster , “Iron Deficiency and Its Role in Cadmium‐Induced Fetal Growth Retardation,” Journal of Nutrition 109 (1979): 1640–1645, 10.1093/jn/109.9.1640. 479957

[60] R. J. Phillips , M. Lutz , and B. Premack , “Differential Signaling Mechanisms Regulate Expression of CC Chemokine Receptor‐2 During Monocyte Maturation,” Journal of Inflammation 2 (2005): 14, 10.1186/1476-9255-2-14. 16259633 PMC1308851

[61] D. Jang , A.‐H. Lee , H.‐Y. Shin , et al., “The Role of Tumor Necrosis Factor Alpha (TNF‐α) in Autoimmune Disease and Current TNF‐α Inhibitors in Therapeutics,” International Journal of Molecular Sciences 22 (2021): 2719, 10.3390/ijms22052719. 33800290 PMC7962638

[62] A. M. Ronco , M. Urrutia , M. Montenegro , and M. N. Llanos , “Cadmium Exposure During Pregnancy Reduces Birth Weight and Increases Maternal and Foetal Glucocorticoids,” Toxicology Letters 188 (2009): 186–191, 10.1016/j.toxlet.2009.04.008. 19379801

[63] M. Kippler , K. Engstrom , S. J. Mlakar , et al., “Sex‐Specific Effects of Early Life Cadmium Exposure on DNA Methylation and Implications for Birth Weight,” Epigenetics 8 (2013): 494–503, 10.4161/epi.24401. 23644563 PMC3741219

[64] M. Kippler , F. Tofail , J. D. Hamadani , et al., “Early‐Life Cadmium Exposure and Child Development in 5‐Year‐Old Girls and Boys: A Cohort Study in Rural Bangladesh,” Environmental Health Perspectives 120 (2012): 1462–1468, 10.1289/ehp.1104431. 22759600 PMC3491924

[65] L. Cheng , B. Zhang , T. Zheng , et al., “Critical Windows of Prenatal Exposure to Cadmium and Size at Birth,” International Journal of Environmental Research and Public Health 14 (2017): 58, 10.3390/ijerph14010058. 28075368 PMC5295309

[66] M. Domellöf , “Meeting the Iron Needs of Low and Very Low Birth Weight Infants,” Annals of Nutrition & Metabolism 71 (2017): 16–23, 10.1159/000480741. 29268255

[67] A. Figueiredo , I. Gomes‐Filho , R. Silva , et al., “Maternal Anemia and Low Birth Weight: A Systematic Review and Meta‐Analysis,” Nutrients 10 (2018): 601, 10.3390/nu10050601. 29757207 PMC5986481

[68] S. A. Weiner , G. Talmon , M. Peniakov , and C. Felszer‐Fisch , “Case 3: Premature Infant With Anemia and High‐Output Cardiac Failure,” NeoReviews 19 (2018): e690–e693, 10.1542/neo.19-11-e690.

[69] G. Salvatori , G. Brindisi , M. Colantonio , and A. M. Zicari , “Cardiac Hypertrophy and Insulin Therapy in a Pre‐Term Newborn: Is There a Relationship?,” Italian Journal of Pediatrics 48 (2022): 24, 10.1186/s13052-022-01216-7. 35135591 PMC8822805

[70] D. Kozlosky , A. Lu , C. Doherty , et al., “Cadmium Reduces Growth of Male Fetuses by Impairing Development of the Placental Vasculature and Reducing Expression of Nutrient Transporters,” Toxicology and Applied Pharmacology 475 (2023): 116636, 10.1016/j.taap.2023.116636. 37487938 PMC10528997

[71] M. E. Romano , D. A. Enquobahrie , C. Simpson , H. Checkoway , and M. A. Williams , “Maternal Body Burden of Cadmium and Offspring Size at Birth,” Environmental Research 147 (2016): 461–468, 10.1016/j.envres.2016.02.029. 26970900 PMC4866807

[72] T. W. Jackson , O. Baars , and S. M. Belcher , “Gestational Cd Exposure in the CD‐1 Mouse Sex‐Specifically Disrupts Essential Metal Ion Homeostasis,” Toxicological Sciences 187 (2022): 254–266, 10.1093/toxsci/kfac027. 35212737 PMC9154225

[73] N. Hegde , M. W. Rich , and C. Gayomali , “The Cardiomyopathy of Iron Deficiency,” Texas Heart Institute Journal 33 (2006): 340–344. 17041692 PMC1592266

[74] Committee on Obstetrics Practice , *Committee Opinion: Lead Screening During Pregnancy and Lactation*, Report No.: 533, Washington, DC (2012). 10.1097/AOG.0b013e31826804e8 22825110

