# Supplementary material for: Dietary Iron Supplementation Protects Against Growth Restriction and Metabolic Dysfunction‐Associated Steatotic Liver Disease in Perinatal Cadmium‐Exposed Mice
Source: FASEB Bioadv. 2025 Jun 17;7(8):e70027. doi: 10.1096/fba.2025-00045 (PMC12312208; doi:10.1096/fba.2025-00045)
Supplement: Supplementary file 1 — Data S1. [file FBA2-7-e70027-s003.docx]

Supplemental Figures

Figure S1. Maternal food consumption during 5-week pre-gestation treatment.

Figure S2. Maternal water consumption during 5-week pre-gestation treatment.

Figure S3. Litter size at time of collection, postnatal day 21 (PND21).

Figure S4. Blood levels of the trace metals magnesium (Mg), manganese (Mn), copper (Cu), selenium (Se), and cadmium (Cd) in (A) males, and (B) females. <LOQ, below limit of quantification. *p<0.05, **p<0.01.
